# Choice of Unmanipulated T Cell Replete Graft for Haploidentical Stem Cell Transplant and Posttransplant Cyclophosphamide in Hematologic Malignancies in Adults: Peripheral Blood or Bone Marrow—Review of Published Literature

**DOI:** 10.1155/2016/6950346

**Published:** 2016-03-28

**Authors:** Shatha Farhan, Edward Peres, Nalini Janakiraman

**Affiliations:** Stem Cell Transplant Program, Henry Ford Hospital, Detroit, MI 48202, USA

## Abstract

Allogeneic hematopoietic stem cell transplantation (SCT) is often the only curative option for many patients with malignant and benign hematological stem cell disorders. However, some issues are still of concern regarding finding a donor like shrinking family sizes in many societies, underrepresentation of the ethnic minorities in the registries, genetic variability for some races, and significant delays in obtaining stem cells after starting the search. So there is a considerable need to develop alternate donor stem cell sources. The rapid and near universal availability of the haploidentical donor is an advantage of the haploidentical SCT and an opportunity that is being explored currently in many centers especially using T cell replete graft and posttransplant cyclophosphamide. This is probably because it does not require expertise in graft manipulation and because of the lower costs. However, there are still lots of unanswered questions, like the effect of use of bone marrow versus peripheral blood as the source of stem cells on graft-versus-host disease, graft versus tumor, overall survival, immune reconstitution, and quality of life. Here we review the available publications on bone marrow and peripheral blood experience in the haploidentical SCT setting.

## 1. Introduction

Allogeneic hematopoietic stem cell transplantation (SCT) is often the only curative option for many patients with malignant and benign hematological stem cell disorders [1]. An HLA-matched related sibling/donor (MRD) is the preferred donor; however, donor availability for many patients still remains a significant challenge as only approximately one-third of patients have an MRD and the shrinking family sizes in many societies are further reducing this probability. The likelihood of identifying a volunteer unrelated donor that is suitably matched at HLA-A, HLA-B, HLA-C, and HLA-DRB1 is population specific ranging from about 79% for Caucasian patients of European descent to 30%–50% for patients of other ethnic backgrounds [2]. This is secondary to the underrepresentation of the ethnic minorities in the registries, significant genetic variability for some races, and expansion of the number of mixed race individuals [3]. In addition, as the age cutoff for reduced intensity conditioning (RIC) and nonmyeloablative (NMA) transplant eligibility has increased, there has been a critical need for alternative donors for those who may not have a suitable HLA-MRD or matched unrelated donor (MUD). Moreover, there are significant delays in obtaining stem cells of couple of months from initiation of the donor search to transplantation [4]. Because high-risk diseases like acute leukemia are more common among the elderly, the time taken to secure a MUD [5] increases the risk of leukemia relapse in this group that needs to proceed to SCT promptly. Even if a matched unrelated donor is identified, the likelihood of proceeding to transplant is less than 50% because of disease progression during the search process [3].

Transplantation from a full haplotype mismatch family donor has been studied for several decades. Potential HLA-haploidentical donors include biological parents or children of a patient, and each sibling has a 50% chance of sharing exactly one HLA haplotype. In most centers, it is possible to identify at least one HLA-haploidentical first-degree relative for more than 95% of patients, and the average number of HLA-haploidentical donors per patient is 2.7 [6]. This rapid and near universal availability of the donor is an advantage of haploidentical SCT and an opportunity that is being explored currently in many centers. However, there are two major historical barriers to a successful haploidentical SCT which include graft rejection and graft-versus-host disease (GVHD) arising from the intense bidirectional alloreactivity and hence a high nonrelapse mortality (NRM) after transplantation. Recently, utilization of different methods to overcome these issues, like the GIAC protocol, pioneered in China, comprising granulocyte-colony-stimulating factor (GCSF) stimulation of the donor; intensified immunosuppression through posttransplantation cyclosporine, mycophenolate mofetil (MMF), and short-course methotrexate; antithymocyte globulin and combination of peripheral blood stem cell and bone marrow allografts; and the use of posttransplantation cyclophosphamide (Cy) [7, 8], and the development of novel methods of selective depletion of T cell subsets, such as the use of *αβ* TCD [9, 10], have improved safety of haploidentical SCT.

Because of lower rate of severe opportunistic infections and less NRM with T replete compared to T cell deplete stem cell transplantation [11, 12] and because T cell depletion is relatively inexpensive and does not require expertise in graft manipulation and the feasibility of posttransplant Cy, T cell replete unmanipulated haploidentical graft is now considered to be a viable alternative option for patients. Posttransplant Cy can induce donor-host tolerance to allografting and decrease GVHD probably by eliminating alloreactive T cell clones without myeloablation [7]. Hematopoietic stem cells are quiescent nondividing cells which express high levels of aldehyde dehydrogenase, likely responsible for cellular resistance to Cy, while T, B, and NK cells express low levels of this enzyme, rendering them sensitive to Cy cytotoxicity [13]. The use of posttransplant Cy has been based on evidence dating back to the 1960s by Berenbaum and Santos who reported that the use of high-dose posttransplant Cy can prevent skin graft rejection when administered 2-3 days after allografting [8]. Immunosuppression after transplant has been shown to promote allograft tolerance and prevent or alleviate GVHD. Storb and colleagues reported that posttransplantation immunosuppression administration with cyclosporine and MMF permits engraftment of major MHC-identical allogeneic bone marrow in dogs with only 200 cGy total body irradiation (TBI) [14]. When this strategy was applied to patients, a 20% incidence of graft failure was noted [15]; this subsequently decreased to 3% after adding a 3-day course of fludarabine to the pretransplant conditioning regimen [16]. The group of Luznik et al. was able to achieve tolerance and multilineage mixed hematopoietic chimerism across MHC barriers in mice conditioned with fludarabine and 200 cGy TBI and given cyclophosphamide 200 mg/kg intraperitoneally on day 2. This regimen was truly NMA as autologous hematopoiesis recovered in mice that were conditioned but did not receive an infusion of marrow [17]. In addition to suppressing graft rejection in sublethally conditioned mice, posttransplant Cy also inhibited GVHD in lethally irradiated mice given MHC-mismatched bone marrow plus a high dose of donor T cells. The administration of cyclosporine or corticosteroids before cyclophosphamide treatment disrupted the tolerance that should be achieved by posttransplant Cy [18, 19] but the tolerance was not affected by the administration of G-CSF starting the day after posttransplant Cy treatment [20]. It was also noted that, in contrast to the conventional GVHD prophylaxis where NRM increase with increasing genetic disparity [21, 22], when using posttransplant Cy, GVHD and NRM were not associated with the degree of HLA mismatching [23].

## 2. Haploidentical Stem Cell Transplantation: Bone Marrow Experience

Researchers from Johns Hopkins Hospital (JHH) reported in 2002 the result of a phase I trial of 13 patients (median age: 53 years) who received a full haplotype mismatched T cell replete bone marrow (BM) graft treated with a NMA regimen consisting of fludarabine 30 mg/m^2^ administered daily for 4 days and 2 Gy TBI followed by posttransplant Cy administration (50 mg/m^2^) on day +3 [24]. The pretransplant conditioning was increased in 10 patients by adding Cy at 14 mg/kg on days −6 and −5 due to an initial higher rate of graft failure noted in the first 3 treated patients. For additional GVHD prophylaxis, MMF and tacrolimus were administered the day after patients received posttransplant Cy (day +4) and continued for at least 30 days. Engraftment was achieved in 8/10 patients in the second cohort (80%), with a median time to absolute neutrophil count >500/microL of 15 days and to unsupported platelet count >20,000/microL of 14 days. All patients with engraftment achieved ≧95% donor chimerism within 60 days of transplantation. Two patients with myelodysplastic syndrome (MDS) rejected their grafts but experienced autologous neutrophil recovery at 24 and 44 days. Grade II–IV acute GVHD developed in 6/13 patients (46%), while grade III-IV acute GVHD developed in only 3/13 patients (23%). After a median follow-up of 6.5 months, 6/10 patients were alive, with 5 remaining in complete remission after transplant [24]. Subsequently, Fred Hutchinson Cancer Center (FHCC) and JHH group published a phase I/II trial of 68 patients who received T cell replete BM haploidentical SCT using Cy 14.5 mg/kg/day on days −6 and −5, fludarabine 30 mg/m^2^/day on days −6 to −2, and 200 cGy TBI on day −1 followed by one or two posttransplant days of 50 mg/m^2^ Cy (day +3 ± day +4) [25]. Twenty-eight patients received one dose and 40 received 2 doses of posttransplant Cy. Tacrolimus and MMF were also used after transplant but tacrolimus was continued until day 180. GCSF support was started on day +1. The median times to neutrophil and platelet recovery were 15 and 24 days, respectively. Graft failure occurred in 9 of 66 (13%) evaluable patients [25]. The cumulative incidence of grades II–IV and grades III-IV acute GVHD was 34% and 6%, respectively. While no significant difference was seen in the incidence of acute GVHD between the two groups, a strong trend towards less extensive chronic GVHD was seen for patients receiving 2 doses as compared with one dose of posttransplant Cy [25]. The cumulative incidence of NRM at day 100 and 1 year was 4% and 15%, respectively. Low rates of NRM, acute GVHD, and chronic GVHD were also reported in a longer follow-up in the expanded cohorts treated in line with the JHH protocol [38]. With 4.1-year median follow-up, 3-year probabilities of relapse, progression-free survival (PFS), and overall survival (OS) were 46%, 40%, and 50%, respectively. On multivariable analyses, the Disease Risk Index (DRI) was statistically significantly associated with relapse, PFS, and OS.

The Blood and Marrow Transplant Clinical Trials Network conducted multicenter phase 2 trials for individuals with leukemia or lymphoma and no suitable related donor. Cyclophosphamide, fludarabine, and 200 cGy of TBI were used with HLA-haploidentical related donor BM transplantation (*n* = 50). The median time to neutrophil and platelet recovery was 16 and 24 days, respectively. The 100-day cumulative incidence of grades II–IV acute GVHD was 32%. There were no reported cases of grades III-IV acute GVHD. The 1-year cumulative incidences of NRM and relapse after haploidentical BM transplantation were 7% and 45%, respectively [27]. The 1-year probabilities of OS and PFS were 62% and 48%, respectively. In this study too, the most frequent cause of death was relapse.

MD Anderson (MDACC) [28, 39], the Italian group [30, 40], and others reported their experience with BM haploidentical SCT using a potentially more ablative conditioning regimen trying to provide more antitumor activity and decrease relapse rate. MDACC used fludarabine, melphalan 100–140 mg/m^2^ based regimen with thiotepa, or TBI 200 cGy. Eighty-four patients had a BM graft except 4 pts. (95%). Overall, for the entire cohort, relapse rate was 32% and PFS was 42.3%. The median OS for first transplants was 25.6 months and it was 6.5 months for second transplant patients. Of the 49 patients who had first transplant for acute myeloid leukemia (AML)/MDS, 27 (55.1%) were in complete remission prior to transplant. NRM for these patients was 9%, relapse rate was 24.3%, and PFS was 66.8% at 50 months of median follow-up [28]. When they compared 32 patients with AML/MDS who received BM haploidentical SCT with MRD and MUD who underwent matched transplantations and received melphalan-based conditioning regimen and conventional GVHD prophylaxis, results were comparable. However, the median time to neutrophil and platelet recovery for haploidentical SCT recipients was 18 and 25 days compared to 13-12 and 14–16 days in MUD and MRD. These differences were probably related to the use of bone marrow stem cells in the haploidentical SCT group [41].

Raiola et al. [30] reported the results of 50 patients with high-risk hematologic malignancies who underwent an unmanipulated haploidentical BM transplant followed by posttransplant Cy. The myeloablative conditioning consisted of thiotepa, busulfan, fludarabine (*n* = 35, 8/35 received reduced dose of busulfan), or TBI 9.9 Gy and fludarabine (*n* = 15). The median age was 42 years (range: 18–66 years); 23 patients were in remission, 27 patients had active disease, and 10 patients were receiving a second allograft. In this study, they used cyclosporine and MMF which were started on days 0 and +1, respectively, in order to better control GVHD, and the second dose of Cy was moved from day +4 to day +5 to decrease the acute toxicity. GCSF was started on day +5. Three patients died before engraftment, and 2 patients had autologous recovery: 45 patients (90%) had full-donor chimerism on day +30. The median day for neutrophil engraftment was day +18. The cumulative incidence of grades II-III acute GVHD was 12%, and that of moderate chronic GVHD was 10%. With a median follow-up for surviving patients of 10.7 months, the cumulative incidence of transplant related mortality (TRM) was 18%, and the rate of relapse was 26%. The actuarial 22-month disease-free survival (DFS) rate was 68% for patients in remission and 37% for patients with active disease (*P* < 0.001). They also published a recent update on 148 patients with encouraging results in terms of engraftment; there was only one patient who developed primary graft failure (0.7%), low rates of GVHD, and transplant mortality. The rate of GVHD, both acute and chronic, was low, and 80% of patients were off cyclosporine at 1 year. Major causes of death were relapse (22%), GVHD (2%), and infections (6%) [40]. When they compared the results of haploidentical SCT to other graft sources including MRD, unrelated donors, and cord, haploidentical SCT grafts were comparable to MRD, whereas UCB had inferior survival [42].

Symons et al. also reported the results of a phase II clinical trial of T cell replete HLA-haploidentical BM transplant using a myeloablative regimen and posttransplant Cy that initially enrolled subjects with refractory hematologic malignancies only, with the later addition of high-risk leukemias in remission and chemosensitive lymphomas. The majority (67%) of patients were not in remission at the time of transplant. Conditioning consisted of IV busulfan (pharmacokinetically adjusted) on days −6 to −3, Cy (50 mg/kg/day) on days −2 and −1 in twenty-seven patients, or Cy (50 mg/kg/day) on days −5 and −4 and TBI (300 cGy/day) on days −3 to 0 in three patients. Donor engraftment at day 60 occurred in all but one evaluable patient (96%, 24/25). The median times to neutrophil and platelet recovery were 25 and 32 days, respectively. The cumulative incidences of grades II–IV and grades III-IV acute GVHD at day 100 were 14% and 7.3%, respectively. The cumulative incidence of chronic GVHD at one year was 13%. The cumulative incidence of NRM at 100 days was 12%. There were no deaths from infection. The cumulative incidence of relapse at 1 year was 66%, in this poor-risk cohort. The cumulative incidence of relapse among patients in complete remission prior to transplant was 13% at 1 year. With a median follow-up of surviving patients of 5.5 months, actuarial OS was 40% at one year. With a median follow-up of event-free patients of 4.5 months, actuarial event-free survival (EFS) was 23.5% at one year [26]. However, disease progression remained a problem in patients with refractory leukemia.

A recent report by the Center for International Blood and Marrow Transplant Research which looked at adults with AML after haploidentical donor (*n* = 192, 162/192 (84%) were BM) and 8/8 HLA-matched unrelated donor (MUD) (*n* = 1982, 1671/1982 (84%) were PB) showed data suggesting that survival for patients with AML after haploidentical transplantation with posttransplant Cy is comparable with MUD. Neutrophil recovery on day 30 after MUD was similar to haploidentical donor in the RIC transplant group, while it was higher in the myeloablative transplant group, and this is probably related to use of BM in most of the haploidentical SCT. In the myeloablative setting, 3-month acute grades II–IV GVHD (16% versus 33%, *P* < 0.0001) and 3-year chronic GVHD (30% versus 53%, *P* < 0.0001) were lower after haploidentical donor compared to MUD. Similar differences were observed after RIC transplants, 19% versus 28% (*P* = 0.05) and 34% versus 52% (*P* = 0.002). Whether the observed low rate of GVHD was solely explained by the use of BM in most of the haploidentical SCT or use of posttransplant Cy or the combination of both cannot be determined. When Ciurea et al. compared chronic GVHD rates in the subset of patients transplanted with BM, there were no differences in 3-year rates of chronic GVHD after haploidentical donor and MUD with myeloablative regimens (30%, *n* = 85 versus 36%, *n* = 231) or with reduced intensity regimens (34%, *n* = 77 versus 30%,  *n* = 80), but these numbers are small and might not show the difference. In addition, 39% of patients who got reduced intensity regimen and 23% of patients who got the myeloablative regimens in the MUD group also received in vivo T cell depletion. Among recipients of reduced intensity regimens, NRM risks were lower after haploidentical compared with MUD transplantation. However, any advantage derived from lower mortality risks with the very low intensity regimen for haploidentical transplantation was negated by higher relapse risks in this group. In the myeloablative setting, an effect of donor type on NRM or relapse risks was not seen. OS was similar between the haploidentical and MUD groups [43].

## 3. Haploidentical Stem Cell Transplantation: Peripheral Blood Experience

Collection of BM stem cells involves the use of the operating room which can be cumbersome, presents an increased risk of complications to the donor, and can make it difficult to reach target CD34 when there is great disparity in weight between the donor and recipient. In addition, in cases of major ABO incompatibility, further time-consuming and complex processing is required. Therefore, there is considerable interest in developing peripheral blood stem cells as a graft source in haploidentical SCT. Recently, emerging data suggest that G-CSF mobilized peripheral blood stem cell (PBSC) graft can also safely be used for haploidentical SCT with posttransplant Cy.

Solomon and colleagues [29, 44] reported the use of busulfan based conditioning regimen with fludarabine and cyclophosphamide (Bu/Flu/Cy) in 20 patients of whom 11 had relapsed/refractory disease. In response to increased rates of mucositis, fludarabine and busulfan doses were decreased by 30% and 15%, respectively, in 15 patients. On day 0, patients received an unmanipulated peripheral blood T cell replete allograft. The cumulative incidence of severe acute GVHD and chronic GVHD was low at 10% and 5%, respectively, with a day 100 NRM of only 10%. For standard-risk patients, NRM was 0% at 100 days and 1 year. The 1-year OS and relapse rates were 69% and 40%, respectively, and were better for standard-risk patients (88% and 33%, resp.). Noninfectious fever (median Tmax 103.9; 101.2–106.8) developed in 18 of 20 patients within a median of 2.5-day (range: 1–5 days) transplantation and resolved in all patients after posttransplant Cy administration. Achievement of full-donor chimerism was rapid with all evaluable patients achieving durable complete donor T cell and myeloid chimerism by day +30. However, they noticed high rates of BK-linked hemorrhagic cystitis (75% of patients) so they published recently the result of Flu/TBI (12 Gy) regimen and PBSC haploidentical SCT [34]. All patients engrafted and achieved sustained complete donor T cell and myeloid chimerism by day +30. When compared with a contemporaneously treated cohort of patients receiving myeloablative HLA-MUD transplantation at their institution, outcomes were statistically similar, with 2-year OS and DFS being 78% and 73%, respectively, after haploidentical SCT versus 71% and 64%, respectively, after MUD transplantation. In patients with DRI low/intermediate risk disease, 2-year DFS was superior after haploidentical compared with MUD transplantations (100% versus 74%, *P* = 0.032), whereas there was no difference in DFS in patients with high/very high-risk disease (39% versus 37% for haploidentical donor and MUD, resp., *P* = 0.821). Rates of grades II to IV acute GVHD were less after haploidentical compared with MUD transplantation (43% versus 63%, *P* = 0.049) as was moderate-to-severe chronic GVHD (22% versus 58%, *P* = 0.003) in spite of the use of PBSC as the stem cell source in all 30 haploidentical transplant recipients compared with 32 of 48 MUD transplant recipients (100% versus 67%, *P* < 0.001). However, GVHD prophylaxis was tacrolimus and methotrexate in all MUD patients, and no patients received in vivo T cell depletion. BK virus-associated cystitis was significantly less frequent after TBI-based myeloablative conditioning with clinically significant hemorrhagic cystitis occurring in only 2 (7%) patients.

Raj et al. published a 4-center experience of 55 patients who underwent T cell replete haploidentical PBSC transplant using RIC followed by posttransplant Cy. The 1-year cumulative incidences of grades II to III acute GVHD were 53% and 8%, respectively. There were no cases of grade IV GVHD. The 2-year cumulative incidence of chronic GVHD was 18%. With a median follow-up of 509 days, OS and EFS at 2 years were 48% and 51%, respectively. The 2-year cumulative incidences of NRM and relapse were 23% and 28%, respectively [31].

Using the same protocol of NMA conditioning regimen, GVHD prophylaxis, growth factor support, and antimicrobial prophylaxis previously reported by Luznik et al. [25], Bhamidipati et al. reported the results of 18 patients who received PBSC haploidentical SCT [32]. Despite the high CD3+ cell dose (median of 19.7 × 10^7^/kg), the cumulative incidence of acute GVHD (all grades) was 41 and 53% on days +60 and +90, respectively. Three patients (17%) developed grades III-IV acute GVHD. The cumulative incidence of chronic GVHD at 1 and 2 years was 8% at both the time points and extensive chronic GVHD developed in only one patient. One-year OS was 62% for all patients and 70% in those patients who underwent transplant in complete remission. Hundred-day and 1-year NRM were 11 and 17%, respectively. The relapse-free survival at 1 year was 53% [32].

More recently retrospective data comparing BM with PB in haploidentical SCT have been reported. Castagna et al. [33] retrospectively looked at the outcome of 2-center haploidentical SCT comparing PBSC and BM in patients with mostly lymphoid malignancies. 46 patients had BM with a median age of 44, while 23 patients had PBSC with median age of 54. They all received FluCyTBI. The incidence of grades II to IV acute GVHD was similar in both groups, 25% and 33% after BM and PBSC infusions, respectively. In addition, chronic GVHD was also similar, 13% after both BM and PBSC infusions. This is probably related to the short term follow-up and the small number of patients which may impair the statistical power of the comparison. No major differences between the 2 cohorts were observed in terms of infectious complications. The relapse incidence was similar in the two cohorts. However, patients in complete remission had a significant lower incidence of relapse (14% versus 33%, *P* = 0.04) and superior PFS (68% versus 49%, *P* = 0.05) compared with those who were not in complete remission. The 2-year overall NRM was 18% (BM: 22%; PBSC: 12%; *P* = 0.96). OS and NRM were not statistically different between the 2 cohorts of patients. They also reported 49 patients with refractory lymphoma (most of them received BM (80%)) who received T-repleted haploidentical SCT with a nonmyeloablative regimen and posttransplant Cy; also in this group the median number of CD34+ cells infused was 3.3 × 10^6^/kg in BM group compared to 5.1 × 10^6^/kg in PBSC group but the median number of days to engraft was similar. Relapse rate was low (18%) [45].

Bradstock et al. [35] compared outcomes for two retrospective cohorts of patients undergoing RIC therapy transplants using haploidentical graft and posttransplant Cy. The graft used was BM in 13 patients and PBSC in 23 patients. Ten of these patients were previously reported [31]. The BM cohort received a single 60 mg/kg dose of cyclophosphamide on day +3, whereas the PBSC cohort received 2 doses on days +3 and +4 and so the 24-month cumulative rates for chronic GVHD were similar in both groups, 28.6% for BM and 32.3% for PBSCs. The 6-month cumulative incidences of acute GVHD were also similar in both groups, 55.1% for BM and 48.5% for PBSCs. Patients in the PBSC group received double the number of CD34+ cells in the stem cell graft; however, times to neutrophil and platelet recovery were not different between the 2 groups. Three patients, all receiving PBSCs, failed to engraft but survived; 2 of these had Philadelphia chromosome positive ALL in first remission, and both recovered with autologous Philadelphia chromosome negative hemopoiesis. The third patient had AML in second remission and because of morbid obesity was unable to receive TBI; he recovered with autologous hemopoiesis. None had significant titers of anti-donor HLA antibodies in their serum, and there is therefore no obvious explanation for this happening in the 2 ALL patients, both of whom had received significant prior chemotherapy. The remaining 33 patients engrafted, with complete donor chimerism documented on DNA testing of blood T cells and granulocytes. The 2-year cumulative incidences of relapse were 43.9% for BM and 23.5% for PBSCs (*P* = 0.286). For the 33 patients with hematological malignancies, the distribution of relapse-free survival did not differ significantly between BM and PBSC groups and at 2 years was 44.9% and 72.7%, respectively. OS at 2 years was significantly better for PBSC patients (*P* = 0.028), at 83.4% versus 52.7% for BM. Patients in the first cohort were slightly older and had a higher proportion of acute myeloid leukemia, but there were no differences in the distribution of DRI scores between the 2 groups. No serious episodes of opportunistic infection occurred in both cohorts and no posttransplant lymphoproliferative disorder was observed.

Another abstract from 14 centers in Spain [36] reported the results of 80 patients (16–66-year-old) who received NMA (77.5%) or myeloablative (22.5%) conditioning regimens and posttransplant Cy with MMF and calcineurin inhibitor. Almost half of the patients (51%) got BM, while the other half (49%) got PBSC. TRM was 19% at 6 months. Grades II–IV acute GVHD was 33% while grades III-IV acute GVHD was 14%. Chronic GVHD was present in 24%, being extensive in 12%. Another multicenter but prospective phase II study was conducted by the Japan Study Group for Cell Therapy and Transplantation [37]. They used a reduced intensity regimen containing busulfan (6.4 mg/kg). GVHD prophylaxis consisted of Cy (50 mg/kg/day on days 3 and 4), tacrolimus (days 5 to 180), and MMF (days 5 to 60). They included large numbers of patients who were not in remission and patients with a history of prior allogeneic SCT compared to other studies. One-year relapse rate was 45% with 1-year DFS and OS rates of 34% and 45%. Grades II–IV acute GVHD was 23%, while grades III-IV acute GVHD was 3%. Chronic GVHD was present in 15%, without any severe GVHD. Subgroup analysis showed that patients who had a history of prior allogeneic SCT (*n* = 13) had lower engraftment (69% versus 100%).

## 4. Conclusions and Future Directions

The studies in Tables 1–3 and others reported over the last decade represent considerable evidence to suggest that haploidentical SCT is a safe and practical option for patients with no donors with almost comparable results to MRD or MUD transplant [38, 43, 44, 46–50] and is superior to conventional consolidation/maintenance chemotherapy as postremission therapy for high-risk diseases [51, 52]. BM has been replaced by PBSC as a stem cell source in MRD and MUD SCT because of the higher engraftment rates due to the larger number of CD34+ stem cells and because of a potential higher graft versus tumor effect linked to a larger number of T cells. In the haploidentical SCT setting, graft rejection rate appears to be similar or slightly lower in most of the studies utilizing PBSC rather than BM as in Table 3. The median days to neutrophils and platelet engraftments appear to be similar between BM and PBSC grafts in spite of higher median CD34 cells in the PBSC grafts. High fever at 4 to 5 days after transplant was observed in both studies with BM or PBSC; however, the median Tmax of patients transplanted with PBSCs was significantly higher than the Tmax of patients transplanted with BM, probably related to high number of T cells [53].

In the study reported by the Blood and Marrow Transplant Clinical Trials Network, chronic GVHD occurred more frequently after PBSC MUD where most patients did not get in vivo T cell depletion, without effect on OS [54], and, in MRD, the higher incidence and greater severity of chronic GVHD in PBSC MRD SCT had little impact on the patient's performance status or survival [55, 56]. Most of the studies that compared haploidentical SCT to MRD or MUD transplants showed less GVHD especially chronic GVHD [41] but it is difficult to tell if this is from the use of BM in most of haploidentical SCT studies or from the use of posttransplant Cy or from both. In the study that compared PBSC haploidentical SCT to MUD SCT [34], rates of acute and moderate-to-severe GVHD were less in haploidentical SCT which may be attributed to use of BM in some of patients in the MUD group and no in vivo T cell depletion or use of posttransplant Cy which is cytotoxic to alloreactive T cells. Interestingly, the number of CD3+ T cells reported in PBSC allografts was about 5-fold higher than the one reported in BM allografts (Table 2) and hence there were higher but acceptable rates of acute GVHD in most of them and similar rates in others (Table 3). Most PBSC studies also showed low rates of severe acute and chronic GVHD and most of them were responsive to steroids. However, most of these studies are from single centers with the small number of patients and short term follow-up especially for the PBSC grafts. The two studies that compared PB with BM [33, 35] are retrospective and small which may impair the statistical power of the comparison. In addition, it is difficult to compare across different trials because of the heterogeneity of patient population and conditioning regimens.

Regarding relapse, the high relapse rate in some of the haploidentical studies compared to other graft sources could be related to the NMA regimen used, use of the BM grafts with low graft versus tumor effect, or lower NRM in haploidentical studies, which puts more patients at risk of relapse. Also effect is probably different depending on the disease too, myeloid or lymphoid malignancies. The effects of the substitution of BM with PBSC in the haploidentical setting on graft versus tumor effect and relapse are also unclear and difficult to assess because of the lack of prospective studies and heterogeneity between the above studies regarding disease risk and regimens used. However, in most of the studies, the most relative factor contributing to outcome was disease risk prior to transplant.

Despite limitations, these studies suggest that BM or PBSC could be safely used as allograft sources for haploidentical transplantation with good outcomes and acceptable rates of GVHD and graft failure, which helps provide more options for patients and donors. However, there is need for prospective adequately powered studies to evaluate the effect of use of BM versus PBSC in haploidentical SCT setting on GVHD, graft versus tumor, OS, immune reconstitution, and quality of life. Since disease relapse or progression remains a problem in high-risk patients, novel therapies added in the conditioning regimens or posttransplant need to be evaluated.

## Figures and Tables

**Table 1:** Platforms of conditioning regimens, GVHD prophylaxis, and graft source.

Reference	Conditioning regimen	GVHD prophylaxis	Graft source
O'Donnell et al., 2002 [24]	FluCyTBI	PTCy D +3, Tac MMF	BM

Luznik et al., 2008 [25]	FluCyTBI	PTCy D +3 ± D +4, Tac MMF	BM

Symons et al., 2011 [26]	BuCy or CyTBI	PTCy, Tac MMF	BM

Brunstein et al., 2011 [27]	FluCyTBI	PTCy, Tac MMF	BM

Pingali et al., 2014 [28]	FluMel Thiotepa or TBI	PTCy, Tac MMF	BM (94%)

Solomon et al., 2012 [29]	FluBuCy	PTCy, Tac MMF	PBSC

Raiola et al., 2013 [30]	FluBu Thiotepa (*n* = 35) FluTBI 9.9 Gy (*n* = 15)	PTCy, CsA MMF	BM

Raj et al., 2014 [31]	FluCyTBI	PTCy, Tac MMF	PBSC

Bhamidipati et al., 2014 [32]	FluCyTBI	PTCy, Tac MMF	PBSC

Castagna et al., 2014 [33]	BM FluCyTBI	PTCy, Tac MMF 74% PTCy, CsA MMF 26%	BM *n* = 46 (67%)
	PBSC FluCyTBI	PTCy, Tac MMF	PBSC *n* = 23 (33%)

Solomon et al., 2015 [34]	Flu/TBI (12 Gy)	PTCy, Tac MMF	PBSC

Bradstock et al., 2015 [35]	BM FluCyTBI	BM PTCy D +3, Tac MMF	BM *n* = 13
	PBSC FluCyTBI	PBSC PTCy D +3 D +4, Tac MMF	PBSC *n* = 23

Gayoso et al., 2013 [36]	NMA 77.5% MA 22.5%	PTCy, CNI, MMF	BM 51% PBSC 49%

Sugita et al., 2015 [37]	FluCyBuTBI	PTCy, Tac MMF	PBSC

BM, bone marrow; Bu, busulfan; CsA, cyclosporine; CNI, calcineurin inhibitor; Cy, cyclophosphamide; D, day; Flu, fludarabine; Mel, melphalan; MMF, mycophenolate; PTCy, posttransplant cyclophosphamide; Tac, tacrolimus; TBI, total body irradiation; PBSC, peripheral blood stem cells.

**Table 2:** Patients, donors, and graft characteristics.

Reference	Pts. number	Med. age (range)	Donors	Disease	Med. CD34 ×10^6^	Med. CD3 ×10^8^
O'Donnell et al., 2002 [24]	13	53	Parent 16% Sib 38% Child 46%	AML/MDS 7 ALL 2 CML 2 MM 1 NHL 1	5.3	0.32

Luznik et al., 2008 [25]	68	46	Parent 28% Sib 48% Child 24%	AML/MDS 28 ALL 4 CML/CMML 6 CLL/NHL 13 HL 13 MM 3 PNH 1	4.8	0.42

Symons et al., 2011 [26]	30	43	NA	AML 16 3 ALL 2 CML 9 NHL	NA	NA

Brunstein et al., 2011 [27]	50	48	Parent 30% Sib 34% Child 36%	AML 22 ALL 9 NHL 12 HL 7	NA	NA

Pingali et al., 2014 [28]	84	46	Parent 15% Sib 42% Child 42% Cousin 1%	AML/MDS 49 ALL 10 CML 9 Lymphoma 13 3 others	NA	NA

Solomon et al., 2012 [29]	20	44	Parent 15% Sib 65% Child 20%	AML 20 ALL 2 NHL 2 HL 1 CML 3	5	1.73

Raiola et al., 2013 [30]	50	42	NA	AML 25 ALL 12 Lymphoma 5 MPD 5 CML 3	4	0.35

Raj et al., 2014 [31]	55	49	Parent 24% Sib 37% Child 39%	AML/MDS 21 ALL 2 NHL 12 HL 9	6.4	2

Bhamidipati et al., 2014 [32]	18	41	Parent 28% Sib 33% Child 39%	AML 12 ALL 2 NHL 2 Other 2	5	1.97

Castagna et al., 2014 [33]	BM, *n* = 46 (67%)	44	NA	AML/MDS 2 ALL 2 HL 23 NHL/CLL 16 MM 2	3	0.34
	PBSC, *n* = 23 (33%)	54	NA	AML/MDS 2 HL 6 NHL/CLL 12 MM 2	5.1	2.73

Solomon et al., 2015 [34]	30	46.5	Parent 7% Sib 40% Child 53%	AML/MDS 17 ALL 6 CML 5 NHL 2	5.01	1.55

Bradstock et al., 2015 [35]	BM, *n* = 13	53	Parent 7% Sib 66% Child 27%	AML 10 NHL 2 CML 1	2.5	NA
	PBSC, *n* = 23	44		AML/MDS 11 NHL 4 ALL 4 Other 4	5.8	NA

Gayoso et al., 2013 [36]	80	37	Parent 35% Sib 44% Child 21%	AML/MDS 30 NHL 5 HL 29 ALL 9 Other 6	NA	NA

Sugita et al., 2015 [37]	31	48	Parent 22.6% Sib 29% Child 45.1% Other 3.2%	AML/MDS 21 ALL 8 NHL 2	4.0	NA

ALL, acute lymphoid leukemia/lymphoma; AML, acute myeloid leukemia; CLL, chronic lymphocytic leukemia; CML, chronic myeloid leukemia; CMML, chronic myelomonocytic leukemia; HL, Hodgkin lymphoma; MDS, myelodysplastic syndrome; MM, multiple myeloma; MPD, myeloproliferative disorder; NHL, non-Hodgkin lymphoma; PNH, paroxysmal nocturnal hemoglobinuria.

**Table 3:** Transplant outcomes.

Reference	Engraf. failure	Med. days to neut./PLT eng.	aGVHD II–IV/III-IV	cGVHD	NRM	Relapse	EFS/PFS	OS
O'Donnell et al., 2002 [24]	20% Cohort 2	15/14	46%/23%	1/10 limited	1/10 died of GVHD	4/10 cohort 2 relapsed	At med. f/u 6 mo. 5/10 in CR	At med. f/u 6 mo. 6/10 (cohort 2) alive

Luznik et al., 2008 [25]	13%	15/24	34%/6%	Ext. 5% in 2 doses of CY versus 25% in one dose of Cy	1 y 15%	1 y 51%	2 y EFS 26%	2 y 36%

Symons et al., 2011 [26]	4%	25/32	14%/7.3%	13%	100 D 12%	1 y 66% poor risk 1 y 13% in CR	1 y 23.5%	1 y 40%

Brunstein et al., 2011 [27]	2%	16/24	32%/0	1 y 13%	1 y 7%	1 y 45%	1 y PFS 48%	1 y 62%

Pingali et al., 2014 [28]	4.7%	18/NA	32.6%/7.8%	21.3%/Ext. 10%	25%	3 y 30.1%	3 y PFS 42.3%	1 y OS 64% Median OS for 1st SCT 25.6 mo. 2nd SCT 6.5 mo.

Solomon et al., 2012 [29]	0%	16/27	30%/10%	35%/severe 5%	1 y 10%	1 y 40%	1 y DFS 50%	1 y 69%

Raiola et al., 2013 [30]	6%	18/23	12%/6%	26%/Ext. 0	6 mo. 18%	18 mo. 22%	DFS 18 mo. 51%	18 mo. 62%

Raj et al., 2014 [31]	4%	17/21	II 35% III 8% IV 0%	18% @ 2 y 2 severe cases	1 y 17%	2 y 28%	2 y 51%	2 y 48%

Bhamidipati et al., 2014 [32]	6%	15/18	All grades 53%/17%	8% @ 2 y Ext. only in 1 patient	1 y 17%	1 y 38%	1 y 53%	1 y 62%

Castagna et al., 2014 [33]	NA	BM 21/29	25%/3%	13%	2 y 19%	1 y 22%	2 y PFS 62%	2 y 68%
	NA	PB 20/27	33%/14%	13%		1 y 12%		

Solomon et al., 2015 [34]	0	16/25	43%/23%	56%/Ext. 10%	2 y 3%	2 y 24%	2 y DFS 73%	2 y 78%

Bradstock et al., 2015 [35]	0	15/18	I–III 55.1% No IV	28.6%	6 mo. 0	2 y 43.9%	2 y EFS 44.9%	2 y 52.7%
	13%	16/24	I–III 48.5% No IV	32.3%	6 mo. 0	2 y 23.5%	2 y EFS 63.6%	2 y 83.4%

Gayoso et al., 2013 [36]	NA	18/27	33%/14%	24%/Ext. 12%	6 mo. 19%	NA	1 y EFS 48%	1 y 60%

Sugita et al., 2015 [37]	13% (0 in no h/o SCT; 31% in h/o SCT)	19/35	23%/3%	15%/none severe	1 y 23%	1 y 45%	1 y DFS 34%	1 y 45%

aGVHD, acute graft versus host disease; cGVHD, chronic graft versus host disease; CR, complete remission; Cy, cyclophosphamide; D, day; DFS, disease-free survival; Engraf., engraftment; EFS, event-free survival; Ext., extensive; mo., months; neut., neutrophils; NRM, nonrelapse mortality; OS, overall survival; PFS, progression-free survival; PLT, platelets; y, year.
